# Understanding the determinants of postnatal care uptake for babies: A mixed effects multilevel modelling of 2016–18 Papua New Guinea Demographic and Health Survey

**DOI:** 10.1186/s12884-021-04318-y

**Published:** 2021-12-23

**Authors:** Francis Appiah, Justice Ofosu Darko Fenteng, Felix Dare, Tarif Salihu, Andrews Ohene Darteh, Matthew Takyi, Patience Ansomah Ayerakwah, Edward Kwabena Ameyaw

**Affiliations:** 1 Department of Population and Health, University of Cape Coast, Cape Coast, Ghana; 2 Berekum College of Education, Berekum, Bono Region Ghana; 3 Department of Optometry, University of Cape Coast, Cape Coast, Ghana; 4 The Australian Centre for Public and Population Health Research (ACPPHR), Faculty of Health, University of Technology Sydney, Ultimo, Australia; 5 L & E Research Consult, Wa, Upper West Region Ghana

**Keywords:** Ecological Model of Human Development, Maternal-level factors, Papua New Guinea, Postnatal care

## Abstract

**Background:**

Papua New Guinea (PNG) recorded 22 neonatal deaths out of every 1,000 livebirths in 2019. Some of these deaths are related to complications that arise shortly after childbirth; hence, postnatal care (PNC) utilisation could serve as a surviving strategy for neonates as recommended by the World Health Organisation. National level study on determinants of PNC uptake in PNG is limited. Utilising the Bronfenbrenner’s Ecological Model of Human Development, the study aimed at assessing determinants of PNC utilisation for babies by their mothers aged 15–49 in PNG.

**Methods:**

The study used data from the women’s file of the 2016–18 PNG Demographic and Health Survey (2016–18 PNGDHS) and a sample of 4,908 women aged 15–49 who had complete information on the variables of interest to the study. Nineteen (19) explanatory variables were selected for the study whereas PNC for babies within first two months after being discharged after birth was the main outcome variable. At 95% confidence interval (95% CI), six multilevel logistic models were built. The Akaike Information Criterion (AIC) was used to assess models’ fit. All analyses were carried out using STATA version 14.0.

**Results:**

Generally, 31% of the women utilised PNC for their babies. Women with primary education [aOR = 1.42, CI = 1.13–1.78], those belonging to the middle wealth quintile [aOR = 1.42, CI = 1.08–1.87], working class [aOR = 1.28, CI = 1.10–1.49], women who had the four or more ANC visits [aOR = 1.23, CI = 1.05–1.43], those with twins [aOR = 1.83, CI = 1.01–3.29], women who belonged to community of medium literate class [aOR = 1.75, CI = 1.34–2.27] and those of moderate socioeconomic status [aOR = 1.60, CI = 1.16–2.21] had higher odds of seeking PNC for their babies. The odds to seek PNC services for babies reduced among the cohabiting women [aOR = 0.79, CI = 0.64–0.96], those at parity four or more [aOR = 0.77, CI = 0.63–0.93], women who gave birth to small babies [aOR = 0.80, CI = 0.67–0.98] and residents in the Highlands region [aOR = 0.47, CI = 0.36–0.62].

**Conclusions:**

Maternal education, wealth quintile, occupation, partner’s education, ANC visits, marital status, parity, child size at birth, twin status, community literacy and socioeconomic status as well as region of residence were associated with PNC uptake for babies in PNG. Variation in PNC uptake for babies existed from one community/cluster to the other. There is the need to strengthen public health education to increase awareness about the benefits of seeking PNC services for babies among women in PNG. Such programs should consider maternal and community/cluster characteristics in their design.

**Supplementary Information:**

The online version contains supplementary material available at 10.1186/s12884-021-04318-y.

## Background

Globally, approximately 6,700 new-borns died every day in 2019 despite reduction in global neonatal deaths from 5.0 million in 1990 to 2.4 million in 2019. Neonatal deaths account for a larger share of under-five deaths due to a faster global decline in mortality among children aged 1–59 months compared to children below one month of life. In particular, in 2019, 47% of all under-five deaths occurred in the neonatal period, and this exceeds what was recorded in 1990 (i.e. 40%). Oceania has experienced remarkable decline of neonatal deaths from 15 deaths per 1,000 livebirths in 1990 to 10 deaths per 1,000 livebirths in 2019. A clear synthesis of Oceania’s neonatal deaths shows that Papua New Guinea is the highest contributor to the sub-region’s neonatal deaths. Typically, whilst Australia’s neonatal deaths reduced from 5 deaths per 1,000 livebirths to 2 deaths per 1,000 livebirths from 1990 to 2019, PNG witnessed a decline of neonatal deaths from 34 deaths per 1,000 livebirths to 22 deaths per 1000 livebirths from 1990 to 2019 [1].

Generally, postnatal period, a stage which marks the first six weeks after birth, is a crucial period in the life of the mother and her new-born [2]. PNC period demands urgent and focused attention in order to avoid preventable morbidity and mortality [3]. Thus, the period provides an avenue for healthcare providers to educate women about healthy breast-feeding practices, screen for postpartum depression, monitor the child’s development and entire health condition, treat delivery related complications, encourage nutrition and insecticide bed net use, and refer mothers and new-borns for dedicated care if required [4, 5]. The WHO, therefore, recommends that mothers and neonates seek early PNC within the first 24 h after delivery and not less than three additional PNC visits within 48–72 h, 7–14 days and 6 weeks after childbirth [6]. However, these guidelines were altered and refined to be context-specific.

As a result, the Government of PNG through its various ministries have implemented a number of strategies/interventions to control maternal and child deaths. Such initiatives include midwifery education, improving access to contraception, emergency obstetric training [7] as well as family and community health care (FCC) through Village Health Volunteers (VHV) and aims at improving maternal, new-born and child health [8]. Again, health policies like the National Health Plan 2011–2020, National Health Service Standards, National Health Sector Gender Policy and National Sexual Reproductive Health policy were also set out to improve maternal and child health service delivery in PNG [9]. Regardless of these political commitments, PNC service utilisation is the most deserted health service in PNG [10]. The 2016–18 PNG DHS indicated that 46% of women reported having received a PNC check-up in the first 2 days after birth whereas only 24% had a postnatal check less than 4 h after childbirth. A substantial fraction of them (51%) did not receive postnatal check at all [10].

Studies have established that, financial constraints, mothers’ attitudes, accessibility of health care facility, interpersonal factors such as husband’s support, parity and pregnancy experiences, mother’s age, place of residence and marital status predict maternal health decision making and healthcare utilisation [11–13]. Plethora of studies have been done on maternal healthcare utilisation in PNG [14, 15]. Davis and colleagues, for instance, noted that factors supporting fathers’ participation in antenatal consultations included feelings of shared responsibility for the unborn child, concern for the mother’s or baby’s health, the child being a first child, friendly health workers, and male health workers [14]. A recent evidence also indicated that rich women, those working and women with secondary or higher education were more likely to utilise skilled attendants during delivery [15].

This is suggestive that, maternal, child, community-level and other factors are critical and play essential role in maternal healthcare utilisation for their babies. However, national level study on the determinants of PNC uptake for babies in PNG is limited. It is against this background that this study seeks to assess the determinants of PNC utilisation for babies in PNG. The study seeks to answer the question, “what factors determine PNC uptake for babies in PNG?” Findings from this study will be useful in designing maternal, child health interventions and campaigns especially those focusing on PNC utilisation for babies.

**Fig. 1:**
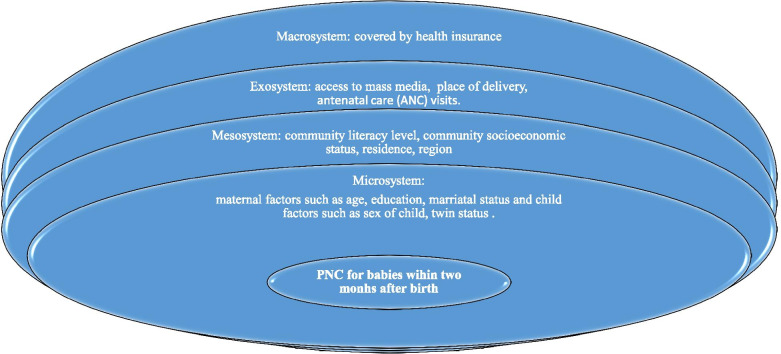
Conceptual Framework for the Study (Bronfenbrenner, 1977)

This study is guided by Bronfenbrenner’s Ecological Model of Human Development (Fig. 1) [16, 17]. Bronfenbrenner conceptualised ecology as a fit between the individual and his/her environment and as such, the fit between the individual and its environment must be even closer in order for an individual to develop and survive [17, 18]. Bronfenbrenner described the ecological environment as composed of systems at four different levels: microsystem, mesosystem, exosystem and macrosystem. The microsystem contains relations between the individual and the immediate environment surrounding the individual, such as the home, school and workplace whereas the mesosystem constitutes interrelations between major settings accommodating an individual, such as relations between home and school, home and peer groups [17, 19].

The exosystem embraces social structures of society including the world of work, the mass media and public agencies. According to Bronfenbrenner, the social structures do not themselves contain the developing person but impinge upon the immediate settings in which that person is found, and as such influence what is going on in these settings [17, 19]. Finally, the macrosystem consists of the blueprints of a particular society such as laws and regulations as well as unprinted rules and norms [17, 20]. Analysing the composition of these ecological systems as well as interactions between and within these systems and individual factors were regarded as crucial in order to understand and explain a developmental outcome [17]. In the context of this study, it is anticipated that maternal and child-level factors and surrounding community-level factors may affect the uptake of PNC. The theory provided a robust means of assessing the determinants of PNC from a wider scope. Figure 1 is the diagrammatical illustration of the framework for the study.

## Methods

### Design and extraction of data for the study

The study adopted a cross sectional survey design and made use of women’s file of the 2016–18 Papua New Guinea Demographic and Health Survey (2016–18 PNGDHS). The 2016–18 PNGDHS is the third in the series of the DHS surveys conducted in the country. The survey was implemented by the National Statistical Office (NSO) of PNG. The survey started data gathering from October 2016 to December 2018. All necessary technical and advisory support were provided by the NSO. The ICF International provided technical assistance through the DHS Program, which offers support and technical assistance for the implementation of population and health surveys.

The survey covered areas such as fertility, family planning, breastfeeding practices, nutritional status of children, maternal and child health, childhood immunisation, adult and childhood mortality, women’s empowerment, domestic violence, malaria, awareness and behaviour concerning HIV/AIDS and other sexually transmitted infections (STIs). The survey applied a stratified sampling technique and in all, a total of 18,175 women aged 15–49 were identified for individual interviews. However, 15,198 women were completely interviewed which yielded a response rate of 84%. Details of the sampling procedures, pretesting of instrument, fieldwork, data processing and analysis can be obtained from the 2016–2018 PNGDHS report [10]. Meanwhile, the present study focused on 4,908 women aged 15–49 who had complete information about the variables of the study.

### Description of study variables

#### Outcome variable

During the 2016–18 PNGDHS, all women who had birth(s) in the 5 years preceding the survey were asked if they had postnatal check-up for their children after exiting the health facilities where they delivered. This was posed as “Did any health care provider or a traditional birth attendant check on (NAME)’s health in the two months after you left?” accompanied by “yes”, “no” and “don’t know” responses. Therefore, the outcome variable for this study was ‘‘postnatal care for babies within first two months after exiting the facility where baby was born’’, defined as having received a postnatal check-up for the baby within first two months after exiting place baby was delivered. Those who affirmed “Yes” were recoded as “1” and “No” recoded as “0”. For precision in responses, “don’t know” responses were excluded from the analysis. Also, the outcome variable excludes pre-discharge checks for babies within facility where births took place to aid assess babies that actually received PNC services after discharge.

#### Explanatory variables

Nineteen (19) explanatory variables were selected for the study [21–23]. These are: age, education, wealth quintile, marital status, occupation, parity, health decision making, partners’ education (maternal factors/microsystem), sex of child, twin status and size of child at birth (child factors/microsystem); community literacy level, community socioeconomic status, residence and region (mesosystem); access to mass media, place of delivery and antenatal care (ANC) visits (exosystem); and covered by health insurance (macrosystem). For clarity of presentation, educational status was recoded into “no education”, “primary”, “secondary/higher”. Occupation recoded into “not working” and “working”; and partner’s education was recoded into “no education”, “primary” and secondary/higher”. Considering the current fertility rate of PNG which is 4.2 children per woman [10], total children ever born was recoded into “one birth”, “two births”, “three births” and “four or more births”. Access to mass media was determined from three principal variables: frequency of reading newspaper/magazine; frequency of listening to the radio; and frequency of watching television which were asked during the 2016–18 PNGDHS. Each of these variables had three responses: ‘not at all’, ‘less than once a week’, and ‘at least once a week’. A composite variable was created whereby all ‘less than once a week’ and ‘at least once a week’ responses were categorised as having access to mass media whilst ‘not at all’ was considered as not having access to mass media. Also, ANC visits were recoded into “less than four visits” and “four or more visits”. For child-level factors, twin status was recoded as “single birth” and “twins” and finally, child’s size at birth recoded as “large”, “average” and “small”. Health decision making was recoded as “alone”, “respondent/partner”, and “others”; place of delivery recoded into “home”, “health facility” and “others”; community literacy level (proportion of women who can read and write at all) was recoded into “low”, “medium” and “high”; and community socioeconomic status was recoded into “low”, “moderate” and “high”. Community literacy level was computed from the women who could read and write at all [24]. Also, community socioeconomic status was measured as the percentage of households in the poorest quintile of Papua New Guinea’s household wealth index [25]. Missing variables were low (3.4%) and were omitted.

### Statistical analysis

The present study assessed determinants of PNC uptake for babies in PNG. Based on the focus of the study, the following steps were involved in analysing the data. The weighting factor inherent in the dataset (v005/100000) and the “svy command” were applied to cater for over and under sampling biases and to account for the complex survey design and generalizability of the findings respectively. Next, computation of women who received PNC for their babies after two months of exiting place baby was born was done (data not shown) and further analysed providers of PNC. Thereafter, a univariate computation of independent variables was done to describe the sample whereas a bivariate analysis was done for the independent variables across PNC utilisation with their chi square test of independence reported. The chi square test of independence helped to gauge independent variables which were not associated with the outcome variable, hence, excluding such variables from the inferential analyses. Also, the “VIF” command was applied to assess collinearity among the explanatory variables and the results indicated no evidence of multicollinearity existing between the explanatory variables (Maximum VIF = 2.55, Minimum VIF = 1.02, Mean VIF = 1.46) (Appendix 1).

Subsequently, at 95% confidence intervals (95% CIs), six (6) multilevel logistic models were built. The first was a null model (Model 0) to account for variability in PNC which can be attributed to the clustering of the primary sampling units (PSUs) without the effect of micro, meso, exo and macro-system. Further, Model I and Model II considered micro and mesosystem-level factors respectively. Model III and IV were fitted to cater for exosystem-level factors and macro system-level factors. Finally, a complete model containing all the factors (Model 0, I, II, III and IV) were constructed (Model V). The results for the fixed effects were presented in adjusted odds ratio (aOR) and any odds less than one was interpreted as reduced likelihood of PNC whilst an odds higher than 1 meant otherwise. Since the models were nested, the Akaike Information Criterion (AIC) was used to measure the model fit [26]. The random effects which are measures of variation of PNC utilisation across communities or clusters, were expressed using Intra-Class Correlation (ICC) and PSUs variance [26, 27]. These were calculated to gauge the variation of PNC utilisation across clusters and the proportion of variance explained by successive models. The entire analyses were done with the aid of STATA version 14.0.

### Ethical considerations

The present study dwelt on an already existing data and since the authors were not involved in the data gathering, no ethical clearance was sought. However, the authors sought for access to use the data from Measure DHS and after obtaining permission, the data was downloaded. The dataset is freely available to the public at https://dhsprogram.com/data/dataset/Papua-New-Guinea_Standard-DHS_2017.cfm?flag=1. However, Measure DHS report has documented details of ethical issues considered in gathering the 2016–18 PNGDHS data [10].

## Results

### Descriptive results for the study

**Fig. 2:**
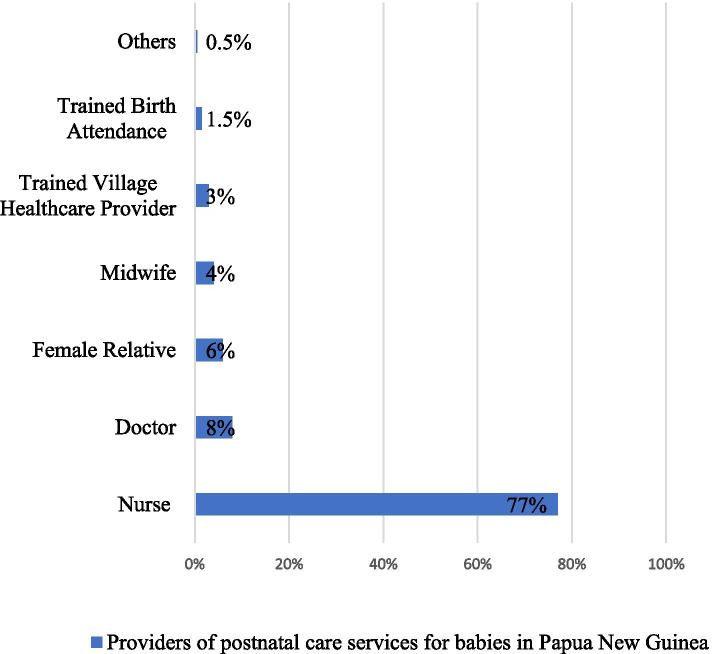
Providers of PNC for babies in Papua New Guinea

Generally, it was revealed that about two-thirds of women aged 15–49 (3,367; 69%) did not receive postnatal services, with only a small fraction of them (1,541; 31%) availing themselves for postnatal check-ups (data not shown). From Fig. 2, most of the women received postnatal check-ups from a nurse (77%).

Table 1 demonstrates descriptive results for the study. It was evident that 35% of women aged 20–24 sought PNC service for their babies. Forty percent of women with secondary or higher education had PNC for their babies. PNC for babies peaked among the richest (43%), the married (34%) and the working class (37%). Women whose partners completed secondary/higher education (38%) topped PNC utilisation for their babies. It was found that women at parity one (36%) and three (36%), those who had access to mass media (40%) and are covered by health insurance (48%) were the highest to seek PNC services for their babies. Women that had four or more ANC visits utilise PNC often (38%) for their babies. PNC utilisation for babies was same among women that gave birth to male (33%) or female (33%) child, just as among women that gave to single or twin babies (33%).

**Table 1:** Descriptive results for the study (*N* = 4,908)

Baby PNC check within 2 months after birth					
**Independent variables**	**Weighted (N)**	**Weighted (%)**	**No (%)**	**Yes (%)**	***X***^***2***^**(*p-value*)**
**Microsystem factors**					
**Age**					4.6448(0.590)
15–19	175	3	69	31	
20–24	1071	22	65	35	
25–29	1311	27	67	33	
30–34	1023	21	67	33	
35–39	800	16	68	32	
40–44	394	8	67	33	
45–49	134	3	74	26	
**Education**					123.6750(0.000)
No education	1218	25	81	19	
Primary	2412	49	65	35	
Secondary/Higher	1277	26	60	40	
**Wealth quintile**					132.9885(0.000)
Poorest	1004	21	81	19	
Poorer	989	20	72	28	
Middle	979	20	67	33	
Richer	981	20	64	36	
Richest	955	19	57	43	
**Marital status**					10.3925(0.001)
Married	4036	82	66	34	
Cohabiting	872	18	72	28	
**Occupation**					26.2159(0.000)
Not working	3268	67	70	30	
Working	1640	33	63	37	
**Partner’s education**					94.4019(0.000)
No education	987	20	81	19	
Primary	2084	43	67	33	
Secondary/Higher	1837	37	62	38	
**Parity**					19.5483(0.000)
One birth	1072	22	64	36	
Two births	987	20	66	34	
Three births	894	18	64	36	
Four or more births	1955	40	71	29	
**Health decision making**					5.5252(0.063)
Alone	1438	29	65	35	
Joint	2752	56	67	33	
Others	718	15	70	30	
**Sex of child**					0.0888(0.766)
Male	2529	52	67	33	
Female	2379	48	67	33	
**Twin status**					6.2002(0.013)
Single birth	4855	99	67	33	
Twin birth	53	1	67	33	
**Size of child at birth**					12.0223(0.002)
Large	1943	39	65	35	
Average	2001	41	67	33	
Small	964	20	71	29	
**Mesosystem factors**					
**Residence**					79.7228(0.000)
Urban	548	11	56	44	
Rural	4360	89	70	30	
**Region**					127.1567(0.000)
Southern region	931	19	57	43	
Highlands region	1868	38	76	24	
Momase region	1429	29	71	29	
Islands region	680	14	65	35	
**Community literacy level**					145.6335(0.000)
Low	2054	42	78	22	
Medium	1471	30	62	38	
High	1383	28	60	40	
**Community socioeconomic status**					119.5434(0.000)
Low	2991	61	74	26	
Moderate	634	13	59	41	
High	1283	26	59	41	
**Exosystem factors**					
**ANC visit**					
Below recommended	2320	47	74	26	72.5628(0.000)
Recommended	2588	53	62	38	
**Access to mass media**					56.3977(0.000)
No	3502	71	70	30	
Yes	1406	29	60	40	
**Place of delivery**					67.1009(0.000)
Home	1924	39	74	26	
Health facility	2838	58	63	37	
Other	146	3	76	24	
**Macrosystem factors**					
**Covered by health insurance**					18.5463(0.000)
No	4755	97	68	32	
Yes	153	3	52	48	

Deduced from 2016–18 PNGDHS

Utilisation of PNC was highest among women that gave birth to large babies (35%). PNC uptake for babies peaked among urban residents (44%), inhabitants of Southern region (43%), women that made health decision alone (35%) and delivered in a health facility (37%). It was found that utilization for babies by women belonging to higher community literacy (40%) topped PNC. Utilisation of PNC for babies peaked among women belonging to moderate (41%) and high (41%) community socioeconomic class. Finally, with the exception of maternal age [*X*^*2*^ = 4.6448, *p* = 0.590], sex of the child [*X*^*2*^ = 0.0888, *p* = 0.766] and health decision making capacity [*X*^*2*^ = 5.5252, *p* = 0.063], the rest of the theoretically selected covariates had significant association with PNC (Table 1).

### Fixed effect results

The Module V is the fixed effect results for study (Table 2). It was evident that, women with Primary education had higher odds to seek PNC for their babies compared with their counterparts without education [aOR = 1.42, CI = 1.13–1.78]. Women in the middle wealth quintile had higher odds to seek PNC for their babies compared with those in the poorest wealth quintile [aOR = 1.42, CI = 1.08–1.87]. It was also revealed that those cohabiting had lower odds to seek PNC for their babies compared with the married [aOR = 0.79, CI = 0.64–0.96]. Comparatively, the results indicated that the working class had higher odds to seek PNC for their babies compared with those who were not working [aOR = 1.28, CI = 1.10–1.49], just as among women whose partners had completed secondary/higher education compared with those whose partners had no education [aOR = 1.32, CI = 1.01–1.72]. Women at parity four or more had lower odds to utilise PNC for their babies compared with those at parity one [aOR = 0.77, CI = 0.63–0.93]. Those that had four or more ANC visits had higher odds to PNC uptake for their babies compared with women who failed to meet the recommended ANC visits [aOR = 1.23, CI = 1.05–1.43]. Women who gave birth to twins had higher odds to seek PNC for their babies compared with those that had single births [aOR = 1.83, CI = 1.01–3.29]. Comparatively, women that gave birth to small babies had lower odds to seek PNC for their babies compared with those that gave birth to large babies [aOR = 0.80, CI = 0.67–0.98]. Residents in the Highlands region lower odds to seek PNC for their babies compared with their counterparts in the Southern region [aOR = 0.47, CI = 0.36–0.62]. Women belonging to community of medium literate class had higher odds to seek PNC for their babies compared with those in the low literate class [aOR = 1.75, CI = 1.34–2.27]. Similarly, women belonging to community of moderate socioeconomic status had higher odds to seek PNC for their babies as compared to those from community of low socioeconomic class [aOR = 1.60, CI = 1.16–2.21].

**Table 2:** A multilevel logistic regression results for the study

Independent variables	Model 0	Model I	Model II	Model III	Model IV	Model V
	**aOR[95%CI]**	**aOR[95%CI]**	**aOR[95%CI]**	**aOR[95%CI]**	**aOR[95%CI]**	**aOR[95%CI]**
**Fixed effects results**						
**Microsystem factors**						
**Education**						
No education		Ref				Ref
Primary		1.63***[1.31–2.04]				1.42**[1.13–1.78]
Secondary/High		1.56**[1.21–2.01]				1.31[0.99–1.72]
**Wealth quintile**						
Poorest		Ref				Ref
Poorer		1.48**[1.13–1.94]				1.40*[1.07–1.84]
Middle		1.59**[1.21–2.09]				1.42*[1.08–1.87]
Richer		1.57**[1.19–2.08]				1.23[0.91–1.65]
Richest		2.00***[1.48–2.72]				1.36[0.95–1.96]
**Marital status**						
Married		Ref				Ref
Cohabiting		0.75**[0.61–0.91]				0.79*[0.64–0.96]
**Occupation**						
Not working		Ref				Ref
Working		1.34***[1.15–1.56]				1.28**[1.10–1.49]
**Partner’s education**						
No education		Ref				Ref
Primary		1.52**[1.19–1.94]				1.36*[1.06–1.73]
Secondary/High		1.45**[1.12–1.89]				1.32*[1.01–1.72]
**Parity**						
One birth		Ref				Ref
Two births		0.81*[0.66–0.99]				0.80*[0.65–0.99]
Three births		0.98[0.79–1.22]				0.97[0.78–1.20]
Four or more births		0.78*[0.65–0.95]				0.77**[0.63–0.93]
**Twin status**						
Single birth		Ref				Ref
Twin birth		1.90*[1.06–3.42]				1.83*[1.01–3.29]
**Size at birth**						
Large		Ref				Ref
Average		0.95[0.82–1.11]				0.97[0.83–1.13]
Small		0.81*[0.66–0.98]				0.80*[0.67–0.98]
**Mesosystem factors**						
**Residence**						
Urban			Ref			Ref
Rural			0.74*[0.55–0.99]			0.76[0.56–1.02]
**Region**						
Southern region			Ref			Ref
Highlands region			0.42***[0.32–055]			0.47***[0.36–0.62]
Momase region			0.54***[0.41–0.70]			0.57***[0.43–0.75]
Islands region			0.62***[0.47–0.81]			0.61***[0.47–0.81]
**Community literacy level**						
Low			Ref			Ref
Medium			2.07***[1.61–2.67]			1.75***[1.34–2.27]
High			1.94***[1.46–2.58]			1.58**[1.16–2.14]
**Community socioeconomic status**						
Low			Ref			Ref
Moderate			1.68**[1.22–2.30]			1.60**[1.16–2.21]
High			1.15[0.86–1.53]			1.07[0.77–1.49]
**Exosystem factors**						
**Access to mass media**						
No				Ref		Ref
Yes				1.31**[1.11–1.54]		0.98[0.81–1.17]
**Place of delivery**						
Home				Ref		Ref
Health facility				1.13[0.94–1.35]		0.84[0.70–1.03]
Other				0.84[0.48–1.46]		0.87[0.50–1.52]
**ANC visit**						
Less **t**han 4 visits				Ref		Ref
4 or more visits				1.40***[1.20–1.63]		1.23*[1.05–1.43]
**Macrosystem factors**						
**Covered by health insurance**						
**No**					Ref	Ref
**Yes**					1.47*[1.03–2.10]	1.09[0.76–1.57]
**Random effect result**						
PSU Variance[95%CI]	1.09[0.87–1.37]	0.85[0.66–1.09]	0.74[0.57–0.96]	0.92[0.72–1.17]	1.07[0.85–1.34]	0.73[0.56–0.96]
ICC	0.25[0.21–0.29]	0.20[0.17–0.25]	0.18[0.15–0.23]	0.22[0.18–0.26]	0.25[0.21–0.29]	0.18[0.15–0.23]
LR Test	311.87***	208.41***	176.70***	230.23***	298.80***	163.97***
Wald X^2^	Ref	134.16***	146.66***	42.73***	4.54*	211.04***
**Model Specification**						
Log likelihood	-2955.449	-2885.5316	-2882.2435	-2934.5971	-2953.1977	-2843.5739
AIC	5914.898	5807.063	5787.487	5881.194	5912.395	5749.148
BIC	5927.895	5924.038	5849.473	5920.186	5931.891	5950.605

*Aor* adjusted Odds Ratio, *CI* Confidence Interval in square brackets, *Ref* Reference Category; *p < 0.05, **p < 0.01, ***p < 0.001

### Random effect results

From Table 2, the random effect results indicated that there was variation in PNC utilisation for babies [σ^2^ = 1.09, CI = 0.87–1.37]. In particular, almost 25% variance for babies in PNC is attributable to variation in the intra class correlation (ICC) (ICC = 0.25). The ICC values reduced in Model II (ICC = 0.18) and Model V (ICC = 0.18). This implies that the variability to utilise PNC for babies is attributable to the variability in the primary sampling units (PSUs).

There were substantial improvement in the desirability of the models specifically in model V (AIC = 5749.148). Therefore, model V is well specified relative to the other models.

## Discussion

The present study aimed at investigating PNC uptake for babies in PNG utilising the Bronfenbrenner’s Ecological Model of Human Development (Fig. 1). The major factors which were associated with PNC uptake were education, wealth quintile, marital status, occupation, partner’s education, parity, twin status, child size at birth (which are found within the microsystem), ANC visits (found within the exosystem) and region, community literacy and socioeconomic status (mesosystem factors). The subsequent section discussed the direction of these key findings.

Consistent with earlier studies [28, 29], the present study noted that women with primary education or belonging to community of medium literate class had higher odds to seek PNC for their babies. This is in line with our framework for the study which indicted that individual level factors also influence their propensity to use health services. Ndugga et al. [21] explained that education enlighten women to be informed and aware of basic health services and health risks which improve their health-seeking behaviour. The authors further argued that higher education empower women to voice their opinions in decisions concerning their own health. Moreover, education exposes mothers to maternal complications and their implications, thereby motivating them to seek timely PNC [30]. This has been corroborated by Yaya et al. [31] who indicated that education affect utilization of all PNC services positively. Congruent with our framework for the study, the study observed that those whose partners had no formal education had lesser odds to seek PNC for their babies. In their meta-analysis, Chaka et al. [32] found that women with husbands who have completed secondary education or above were more likely to use PNC service than those whose husbands had informal or no education in Ethiopia. It is argued that educated husbands are knowledgeable about the benefits of PNC uptake and hence less restrictive towards their wives’ uptake of PNC services [32, 33].

In agreement to previous study [32], the present study found that neonates born by those in the middle wealth quintile or belonging to community of moderate socioeconomic status had higher odds to seek PNC. Maternal wealth status has the potential to influence their PNC utilisation since mothers with enough funds can afford user fees associated with PNC services as highlighted by the framework of the study. Chaka and colleagues [32] found that women with middle household monthly income were more likely to use PNC service than those with lower monthly income in Ethiopia. A plausible reason given in the literature is that wealthier women belong to households that can afford medical and non-medical cost of PNC [32–34]. Practically, reproductive healthcare services are provided across aid posts, health sub centres, health centres, district hospitals and provincial public hospitals, in ascending order of size and resources in PNG which are either publicly or privately owned [35, 36]. Despite the availability of these services, there is low uptake of healthcare services among infants and children by their caregivers in PNG, with significant income-related inequalities in use [37]. Also, most caregivers in PNG tend to rely on healthcare services for their infants and children provided by non-hospital public facilities such as health centres and government aid posts, public hospitals and church health facilities. This is because, some care givers especially the poor, are unable to afford user-fees associated with utilization of essential healthcare services particularly, those provided by private-owned facility [37]. Therefore, our observation is not surprising.

In relation to the above, residents in the Highlands region had lower odds to seek PNC for their babies compared with their counterparts in the Southern region. Region of residence, considered in our study as a mesosystem factor (Fig. 1), plausibly influence PNC uptake in the sense that mothers, especially those distant from a health facility, have to walk for longer distance before reaching a facility to assess PNC services for their babies. In particular, PNG, considered as one of the most rural countries in the world, is also characterised by isolated populations with four out of five people living in rugged or coastal terrain without access to roads and public transportation [38]. Bauze et al. [39] added that restricted or poor access to roads and public services in rural areas is a major obstacle to accessing health care in PNG especially among women and children in rural areas [39]. Izudi et al. [40] further argued that long distances limit the willingness and ability of postpartum women to seek PNC due to the physical difficulties of travel and high costs of motorized transport. Probably, women in the Highland areas have to travel over a longer distance in accessing health facility for PNC services.

The present study noted that the cohabiting had lower odds to seek PNC for their babies compared with the married which supports the framework used for the study (Fig. 1). Plausibly, partner or husbands’ support including financial assistance rendered to their spouse could enrich the married and enable them afford user fees associated with PNC utilisation [17, 19]. It is increasingly known that unemployed women are more likely to be economically dependent and consequently unable to access and use maternal health services [41, 42]. Therefore, observing that the working class have higher odds to utilise PNC for their babies in PNG in the present study is not surprising as such women are economically independent and can afford user fees associated with PNC utilisation. In Nigeria, Adigun and Adigun [43] found that women belonging to the working class used high quality PNC services than non-working class. Ndugga et al. [21] also found that unemployed women had lower odds of PNC within 2 days after childbirth compared with women employed in the agricultural sector in Uganda.

Women at parity four or more had lower odds to utilise PNC for their babies compared with those at parity one. Probable reason could be that women of higher parity might depend on their experience acquired from previous PNC visits. On the other hand, it is held that multiple demands including caring for new-borns and older children often prohibit women from seeking PNC particularly where proximity to a health facility is perceived to be a challenge [21]. Any of these explanations could accounts for our observation in the present study. Additionally, those that had four or more ANC visits had higher odds to PNC uptake for their babies compared with women who had less than four visits. The exosystem which embraces social structures of society including health facilities where mothers receive ANC services (Fig. 1), could exposed mothers to useful health information such as the benefits of using PNC services for their babies; hence, influence their PNC uptake for their babies. The results are in line with a study by Fekadu and colleagues [44] who found that mothers who received more ANC components had higher odds of also receiving PNC in Ethiopia. Fekadu and co explained that receiving more components of ANC implies that the mother is more likely to be informed about complications that may occur after delivery which encourages them to seek timely postnatal care [44]. Similar reason could explain our phenomenon.

Women who gave birth to twins had higher odds to seek PNC compared with those that had single births. PNC service utilisation is essential since such services give health staff platform to detect and manage maternal and new-born diseases. As such, women need to avail themselves for such services devoid of type of birth. This confirms our framework used for the study (Fig. 1). Perhaps, perception held about giving birth to twins encourages women to seek PNC for twin births, however, further studies on giving birth to twins and PNC uptake is needed to understand the phenomenon better. Finally, the present study catered for the hierarchical structures embedded in the sampling design of PNG DHS and realised that variation in community characteristics accounted for variation in PNC uptake in PNG. As a result, interventions targeted at increasing PNC uptake for babies in PNG must take into consideration the inequalities that exist among communities which influence PNC utilisation.

### Strengths and weaknesses

The main strength of this study is that the data originated from a current nationwide survey that drew respondents using probabilistic methods. In addition to larger sample size, the study employed a rigorous analytical procedure and also accounted for community/cluster influence on PNC utilization which improved the robustness of results. Also, sampling errors were controlled using the weighting factor inherent in the data (v005/100000). However, the results of the present study should be interpreted with caution. Firstly, causality cannot be established since the study depended on data derived from cross-sectional survey design. Secondly, the study is not free from social desirability and recall biases. Additionally, the study design limited the effort to unravel reasons to explain some of the observed findings. Additional limitations could include validity of survey questions. Also, this study relied on the framework which shows that there are ecological factors associated with PNC utilisation; yet, structural changes in the availability, affordability and accessibility of PNC are crucial. Although these factors are not the focus of the paper.

## Conclusions

In conclusion, maternal education, wealth quintile, occupation, partners’ education, marital status, parity, child size at birth, twin status (microsystem), community literacy, socioeconomic status, region of residence (mesosystem factors) as well as ANC visits (exosystem), are positively associated with PNC uptake for babies in PNG. Additionally, variation in PNC uptake exist across communities/clusters. These significant findings have policy implications and as such, the following recommendations are needful. Firstly, female education should be a priority in PNG whereas barriers to education faced by females should be addressed by the Government of PNG through its education ministry. Secondly, there is an urgent need for public health education to increase awareness about the benefits of seeking PNC services for babies among women in PNG. Such programs should consider maternal and community characteristics in its program design.

## Supplementary Information

Additional file 1: Multicollinearity testing.

## Data Availability

The datasets generated and/or analysed during the current study are available in the Measure DHS repository, http://www.measuredhs.com.

## Abbreviations

ANC: Antenatal Care
AIC: Akaike Information Criterion
AOR: Adjusted Odds Ratio
FCC: Family and Community Health care
GOPNG: Government of Papua New Guinea
ICC: Intra-Class Correlation
ICF: Inner-City Fund
MMR: Maternal Mortality Ratio
NSO: National Statistical Office
PNG: Papua New Guinea
PNC: Postnatal Care
PNGDHS: Papua New Guinea Demographic and Health Survey
PSU: Primary Sampling Units
STIs: Sexually Transmitted Infections
DFAT: The Australian Government Department of Foreign Affairs and Trade
UNFPA: The United Nations Population Fund
UNICEF: The United Nations Children’s Fund
VHV: Village Health Volunteers
