# Supplementary material for: Understanding the determinants of postnatal care uptake for babies: A mixed effects multilevel modelling of 2016–18 Papua New Guinea Demographic and Health Survey
Source: BMC Pregnancy Childbirth. 2021 Dec 23;21:841. doi: 10.1186/s12884-021-04318-y (PMC8697438; doi:10.1186/s12884-021-04318-y)
Supplement: Supplementary file 1 — Additional file 1: Multicollinearity testing. [file 12884_2021_4318_MOESM1_ESM.docx]

**Appendix 1: Multicollinearity testing**

| Variable | VIF | 1/VIF |
| --- | --- | --- |
| Community Socioeconomic status | 2.55 | 0.391675 |
| Wealth quintile | 2.44 | 0.409743 |
| Community literacy level | 1.90 | 0.527297 |
| Education | 1.79 | 0.559813 |
| Residence | 1.74 | 0.573764 |
| Partner’s education | 1.52 | 0.655837 |
| Access to mass media | 1.49 | 0.671446 |
| Place of delivery | 1.32 | 0.756586 |
| ANC visit | 1.21 | 0.827133 |
| Region | 1.10 | 0.907842 |
| Covered by health insurance | 1.10 | 0.911030 |
| Parity | 1.08 | 0.929403 |
| Occupation | 1.07 | 0.931752 |
| Size of child at birth | 1.05 | 0.950473 |
| Marital status | 1.03 | 0.967819 |
| Twin status | 1.02 | 0.982498 |
| Mean VIF | 1.46 |  |
